# How the Spouse's Retirement Affects the Cognitive Health of Individuals in China: A Fresh Evidence From the Perspective of Social Interaction

**DOI:** 10.3389/fpubh.2021.796775

**Published:** 2021-12-16

**Authors:** Xiaohan Xiong, Rui Li, Hualei Yang

**Affiliations:** School of Public Administration, Zhongnan University of Economics and Laws, Wuhan, China

**Keywords:** social interaction, spouse's retirement, cognitive health, spillover effect, dual-earner couples

## Abstract

**Background:** With the rapid aging of global population, the health consequences of retirement reform are debated greatly. However, most previous studies are limited to the effects on individual themselves, and pay scant attention to the social interaction between individuals and their spouse which may induce the social multiplier effect of retirement. Driven by the practical and academic motives, this study investigates the impacts of the spouse's retirement on the individual's cognitive health among Chinese dual-earner couples.

**Methods:** We first build a simultaneous-equations model. Then, using the data from the 2010 to 2018 China Family Panel Studies (CFPS), we choose the fixed-effects model and adopt the fuzzy regression discontinuity design method to analyze. Besides, we check the validity and robustness of the results. Finally, we employ the mediating effect model to explore the mechanisms.

**Results and Conclusions:** The spouse's retirement has significantly negative direct and indirect effect on individual cognitive health. Husbands' retirement has a stronger adverse spillover effect than wives' retirement, and wives' cognitive health is more vulnerable to the social interaction effect. The direct spillover effect of husbands' retirement is −0.503 and that of wives' retirement is −0.312, the indirect spillover effect of husbands' retirement is −0.36 and that of wives' retirement is −0.279. In addition to the social interaction effect of cognition between the couples, we also find that the decrease in household income is an important mechanism, and that the increased exercise frequency can somewhat mitigate the adverse spillover effect.

## Introduction

Due to the increasing life expectancy and a declining birth rate, the proportion of elders around the globe continues to rise rapidly. Among all the major countries, China especially is facing the largest and fastest growth in population aging. According to the report of National Bureau of Statistics of China 2020, the population of those above 65 took up 12.6% of the total population, and the elderly dependency ratio was 17.8% in 2019. The rate of aging in China already reaches 49.6% within the decade. Aging population tends to be accompanied by a significant drop in labor force participation. As a consequence, China will raise the statutory retirement age.

There is a growing consensus that retirement reforms, especially changes in retirement age, should carefully account for adverse effects on health, which may, in turn, affect long-term care expenditures (1). Several recent prospective studies on workers' cognitive health trajectories prior and after transition to retirement seem to suggest that retirement could be harmful to cognitive health, but the evidences are inconsistent. Based on the hypothesis of “(cognitive ability) use it or lose it,” Rohwedder and Willis used cross-nationally comparable survey data and the instrumental variable method, and found the significant negative impact of retirement on cognitive ability (2). However, Bingley and Martinello stated reasons to be skeptical of this method and its findings (3). They argued that there might be selection bias due to education level and career choice are not controlled. After the two important variables are controlled, most studies conclude that retirement accelerates cognitive decline [e.g., (4–6)]. A few studies showed that retirement decreases cognition for most workers, but improves cognitive health for blue-collar workers (7).

The above literature ignores the impact of an individual's retirement on the cognitive health of significant others. In fact, individuals are bound to be influenced by the events that occur in the life course of those around them (8), because individuals are nested in specific social relationships rather than live a life without connecting with others. In society, the most basic unit is the family. The conjugal relation is the core of the family, and also the most intimate relationship in society (9). Couples share intra-household resources such as household income and living room with each other and take on corresponding domestic responsibilities and affairs with different roles. Therefore, it is clear that retirement sets in motion a sequence of events that have interaction effects with their spouse and thus may affect the cognitive health of their spouse. Individual retirement affects not only their own cognitive health but also those of their spouse, which we refer to as “the spousal spillover effect.”

During the accelerated transformation of Chinese society, women's labor force participation rate has been increasing, and dual-earner couples have become the dominant form of modern families (10), which means that retirement hits most families twice. However, a considerable number of studies only focus on the impact of individual retirement but overlook the shock from the spouse's retirement. If the spousal spillover effect of retirement exists, there would be a social multiplier effect of the retirement policy, and the previous studies could largely underestimate the influence of delaying the statutory retirement age. Hence, as an essential element, the spillover effect of the spouse's retirement on individual cognitive health should be taken into account when calculating the total retirement effect.

It is surprising that such spousal spillover effect has not received much attention in the literature. At present, the relevant papers are no more than 10, and mainly explore the spillover effects of the spouse's retirement on individual physical and mental health. The conclusions are not yet consistent. They can be divided into three views. (i) The spouse's retirement improves individual health. Using the data from Australia, Atalay and Zhu analyzed and found that wives' retirement has a positive effect on husbands' mental health, and this positive effect increases with the duration of wives' retirement (11). Zang used 2011, 2013, and 2015 data from the China Health and Retirement Longitudinal Study (CHARLS), and found that the retirement of husbands led wives to increase the frequency of socialization and exercise, thereby improving wives' physical and mental health (12). (ii) The spouse's retirement declines individual health. Bertoni and Brunello used Japanese panel data to examine “Retired Husband Syndrome” and showed that the husband's retirement causes the higher economic distress, and thus reduced the wife's mental health (13). Müller and Shaikh used panel data from 19 European countries to study the impact of spousal retirement on individual health behavior. The authors found that spousal retirement led individuals to reduce physical activity and increased alcohol consumption, which contributed to reducing the individuals' self-rated health (14). (iii) The spousal spillover effect is heterogenous by gender. Xiong and Li used CFPS data and the results showed that retirement increased husbands' self-rated health by 28-44.3% and decreased wives' self-rated health by approximately 32% (15). As we can see, compared to previous studies which treat retirees as isolated individuals, these studies breakthrough the “Stable Unit Intervention Value Assumption (SUTVA)^1^.”

Up to now, far too little research has been carried out from the perspective of social interaction. As for social interaction, Manski took the lead in strictly defining it and divided it into three categories (17): (i) endogenous effects, in which an individual's propensity to behave in some way differs with the group's behavior; (ii) exogenous (contextual) effects, in which an individual's propensity to behave in some way varies with the group's exogenous character traits; and (iii) correlated effects, in which individuals in the same group tend to behave similarly because they have similar individual characteristics or face similar institutional environments. Furthermore, Manski pointed out that the linear social interaction model which was used to estimate endogenous effects suffers from the reflection problem. To solve the reflection problem, more and more recent scholars use the method of combining the instrumental variable method and the simultaneous-equations model [e.g., (18, 19)].

This study gives a fresh perspective of social interaction to investigate how the spouse's retirement affects an individual cognitive health. More specifically, this study seeks to examine the hypothesis that whether there is a direct spillover effect and an indirect spillover effect of the spouse's retirement on the individual cognitive health. The direct spillover effect happens through the changes caused by the spouse's retirement in the household income per capita and the share of housework undertaken by the wife. And the indirect spillover effect corresponds to the endogenous effect, that is, the social interaction effect of cognitive ability between the couples after the spouse retires.

Data from the 2010-2018 China Family Panel Studies (CFPS) are used in this paper. The sample is restricted to the dual-earner couples with husbands aged 50–70 and wives aged 40–60. Constructing a simultaneous-equations model, this paper adopts the fuzzy regression discontinuity design (FRD) method to identify the direct spillover effect and indirect spillover effect of the spouse's retirement on the individual cognitive health. Furthermore, to explain why the spousal spillover effects occur, the study also analyzes the mediating effects of social interaction between the couple's cognition, family resources (including household income per capita, the share of housework undertaken by the wife) and health lifestyle (including cigarette amounts a day, whether to drink alcohol frequently, and exercise frequency).

## Institutional Background

### Statutory Retirement Age in Urban China

The statutory (full) retirement age in China is 60 years for men, 55 years for female civil servants, and 50 years for other female employees. China has the lowest retirement ages in the world, even though its population is aging fast as a result of birth control policies and increasing life expectancy. For historical reasons, statutory (full) retirement ages only apply to urban China.^2^ Retirement arrangements were introduced to protect urban employees in the 1950s when the only employers were either the government or state-owned companies and institutions. Private sector and self-employment entered after the economic reforms in the 1980s. Retirement arrangements were adapted to cover urban workers in these “new” sectors, but still do not apply to rural China. Farmers usually continue working as long as their health permits. In this study, we therefore restrict our analysis to urban workers.

In principle, employees are required to retire at their statutory retirement age, but deviations are possible: (1) Employees are allowed to retire 5 years earlier than the full retirement age if their jobs are dangerous or harmful to health, or if a medical exam proves that they are too ill to continue working. (2) Retirement at the statutory retirement age is not as strictly enforced in the private sector, self-employment, and temporary employment as in the public sector and state-owned companies. Therefore, “compliance” with the statutory retirement age is not perfect: a substantial number of people still works for pay after reaching the statutory retirement age. This is the reason why we adopt the FRD method.

### Pension and Processed Retirement

Urban employees are required to participate in pension programs. This policy is strictly enforced in the public sector, state-owned enterprises, and big companies in the private sector. Deviations exist in small private companies and in informal employment.

Employees are eligible to claim a pension when they reach their statutory retirement age and “process” retirement. The pension income varies in amount and composition, depending on pension program, years of contribution, and occupation. The actual pension income can be lower or higher than the pre-retirement wage.

“Processed retirement” means that an employee reaching the statutory retirement age leaves the current job after going through all the formalities with employer and local government. A difference from many other countries is that people can still continue working after “processing retirement.” They can work for a new employer or even for the former employer with a temporary contract, while at the same time claiming pension (and health insurance benefits) from the former employer. This fact complicates the definition of retirement, which we will further discuss in section Materials and Methods.

## Materials and Methods

### Data Sources and Sample

The data used in this paper are from the 2010 to 2018 CFPS. The CFPS is a biennial longitudinal survey conducted by the Institution of Social Science Survey at Peking University. This investigation launched in 2010 with five waves of publicly released datasets. The samples covered 25 provinces, accounting for 95% of the total population of China. The contents of CFPS are rather typical, covering the demographics, socioeconomic condition, education, and health of respondents.

**Figure 1:**
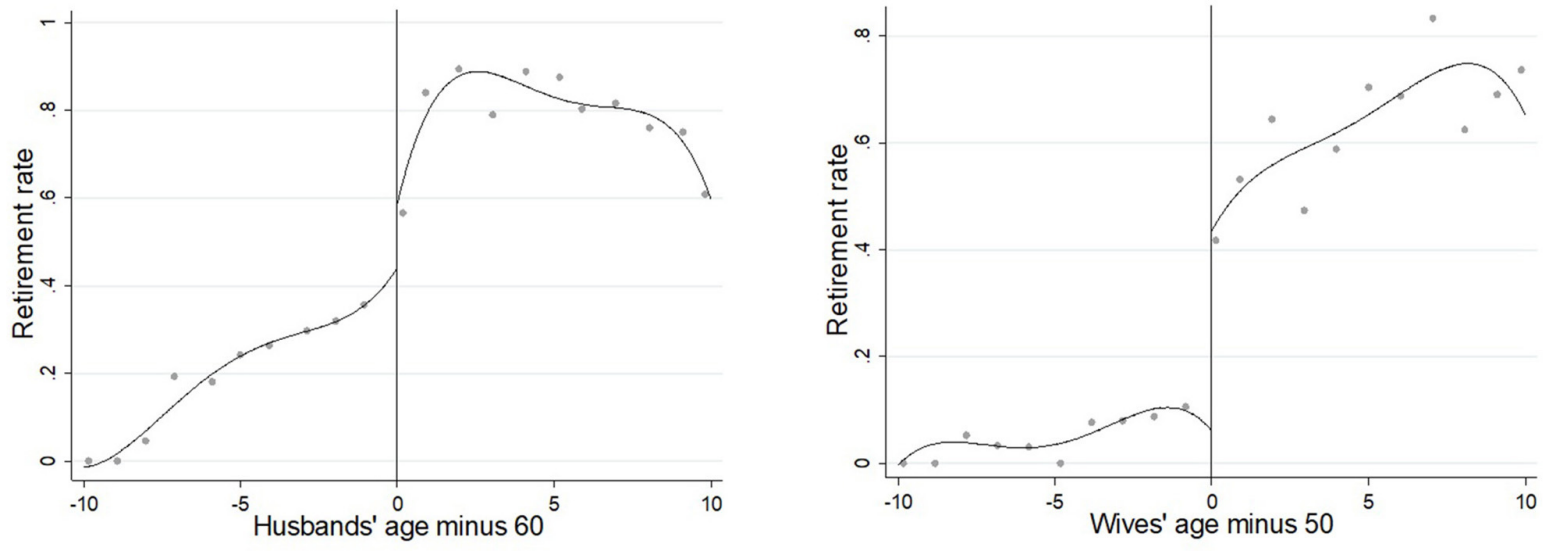
Retirement rate by age among dual-earner couples. The vertical lines at ages 50 and 60 are the statutory retirement ages for female and male workers.

We restrict the sample to the respondents who are married to a surviving spouse. This reduces the sample size from 411,130 to 286,789 individual-year observations. We further exclude the couples where at least one person is not in pension programs since they do not process retirement. The sample size is reduced to 25,752. Then, based on the statutory retirement age cutoff points (see Figure 1), we restrict the sample to couples with husbands aged 50-70 and wives aged 40-60. Intuitively, the closer the age range is to the statutory retirement age, the more precise the sample is; however, as the age range becomes narrower, it leads to a smaller number of observations. Therefore, to allow a reasonable estimate, we preserve 10 years on either side of the statutory retirement age. In the later stages of this study, we will use narrower age ranges to check robustness. In this step, we obtain a sample of 10,663. Finally, we exclude observations with missing information on explained or explanatory variables, leaving us with a sample of 10,599 individual-year observations for 4,107 individuals.

The Biomedical Ethics Review Committee of Peking University approved CFPS, and all participants were required to provide written informed consent. The ethical approval number was IRB00001052-14010.

### Variables

#### Explained Variable: Cognitive Health

Cognitive health refers to brain's ability to process information, such as memory, numeracy, fluency, orientation, logic, reaction and so on. It should be carefully distinguished from the mental health, which is more related to individual's happiness, confidence, resilience etc. Depending on the transition over lifecycle, cognition has been commonly classified into crystallized cognition and fluid cognition (21). While crystallized cognition remains fairly stable over life cycle, fluid cognition has a clear declining pattern as people age.

CFPS database asks about crystallized cognition (verbal tests and math tests) in 2010, 2014, and 2018, and fluid cognition (word recall tests and number series tests) in 2012 and 2016. CFPS data has raw scores ranging from 0 to 34 for verbal tests, 0-24 for math tests, 0-10 for word recall tests, and 0-15 for number series tests. In order to make the estimates more comparable, this paper converts, respectively, the scores of the four tests into a standardized score with a mean of 0 and a standard deviation of 1. Finally, the average of the four standardized scores is the value of cognitive health.

#### Explanatory Variables: Spouse's Retirement and Individual Retirement

Throughout the paper, the key independent variable is the retirement status of individuals and their spouse, which is defined as both “processed retirement” and not working for a paid job anymore. It is constructed based on the following questions: First, the respondent is asked “Are you presently working?”^3^ Those who answer “yes” are included in the control group; those who answer “no” are further asked “Why are you not working?” The answers to this question include “seeking work,” “doing housework,” “disabled,” “student,” “retired,” “other” and “unknown.” Those who answer “retired” are included in the treatment group, otherwise are in the control group. As a result, our retirement status variable equals 1 if the respondent is in the treatment group and zero if he or she is in the control group.

#### Control Variables

Ages of individuals and their spouse. Figure 1 shows the “retirement rate” (the sample fraction of husbands and wives in retirement) by age and gender. It shows a clear discontinuity at age 60 for husbands and at age 50 for wives. These are related to formal retirement rules.

For female civil servants, the statutory retirement age is 55. In Figure 1, we do not see a clear discontinuity in retirement rates for wives at age 55, since civil servants are a relatively small group, and the distinction between civil servants and other public sector employees is poorly measured in our data. We therefore proxy the statutory retirement age to age 50 for wives.

In addition, there are six predetermined variables. They are urban-hukou, education level, family size, possess more than one house, participated in medical insurance, and mainly cared for by spouse in case of illness. Education level is categorized as Illiterate/semi-literate, Primary and middle school education (finished primary school or middle school), High school and above education (finished high school/polytechnic school/technical school/vocational high school, junior college, bachelor's degree, master's degree, and doctor's degree). All of the above predetermined variables are binary variables except for family size, for which a value of 1 means “yes” and a value of 0 means “no.”

#### Mechanism Variables

This paper examines three mechanisms through which the spouse's retirement may affect the individual's cognitive health: social interaction, family resources, and health lifestyle. Specifically, the three mechanisms include six variables: the spouse's cognitive health, household income per capita, the share of housework undertaken by the wife, cigarette amounts a day, whether to drink alcohol frequently and exercise frequency.

According to the theory about social interaction, there may be an endogenous effect of cognitive health between the couples after retirement. It means that the spouse's retirement may affect the individual's cognitive health through the spouse's own cognitive health. We refer to this pathway as the social interaction mechanism and to the effect it generates as the indirect spillover effect. The proxy variable of social interaction mechanism is the spouse's cognitive health.

Retirement leads directly to a decrease in household income. The reduction in household income can cause the couples to invest less in their health (22), and may negatively affect the couple's cognitive health. Retirement also affects both spouses' household contributions (23). Upon retirement, couples may re-negotiate the division of housework, and the share of housework undertaken by the wife may be decreased (12). Changes in the division of housework can affect the spouse's leisure time and the probability that the spouse will engage in activities that benefit their cognitive health. Hence, the spouse's retirement may affect the individual's cognitive health through household income and division of housework, both of which are family resources. In this paper, we use household income per capita and the share of housework undertaken by the wife to examine.

In addition, as a proximal factor in health, lifestyle is an important mechanism that has been examined by many scholars in the past (24, 25). In this paper, we examine the effect of the spouse's retirement on the following aspects of the individual's health lifestyle: cigarette amounts a day, whether to drink alcohol frequently (drink at least three times a week), and exercise frequency.

### Model Settings

#### Setting the Fuzzy Regression Discontinuity Design Model

Based on the characteristics of panel data from the CFPS data base, the following simultaneous-equations model is constructed to verify the spillover effect of the spouse's retirement on individual cognitive health:


(1) $$\begin{array}{l} \{\begin{array}{c} C_{i,t}^{m}=\beta_{0}^{m}+\beta_{1}^{m}R_{i,t}^{m}+\beta_{2}^{m}R_{i,t}^{f}+\beta_{3}^{m}C_{i,t}^{f}+\beta_{4}^{m}X_{i,t}^{m}+\mu_{i}^{m}\\ +v_{t}^{m}+\varepsilon_{i,t}^{m}\\ C_{i,t}^{f}=\beta_{0}^{f}+\beta_{1}^{f}R_{i,t}^{f}+\beta_{2}^{f}R_{i,t}^{m}+\beta_{3}^{f}C_{i,t}^{m}+\beta_{4}^{f}X_{i,t}^{f}+\mu_{i}^{f}\\ +v_{t}^{f}+\varepsilon_{i,t}^{f} \end{array} \end{array}$$

where *m* denotes the husband and *f* denotes the wife. $C_{i,t}^{m}$ denotes the cognitive health of the husband in household *i* and year *t*, and $C_{i,t}^{f}$ denotes the cognitive health of the wife in household *i* and year *t*. $R_{i,t}^{m}$ denotes the retirement status of the husband and $R_{i,t}^{f}$ denotes the retirement status of the wife. $X_{i,t}^{m}$ is a vector of observable covariates affecting the husband cognitive ability, $X_{i,t}^{f}$ is a vector of observable covariates affecting the wife's cognitive health. μ_*i*_ is the individual effect which does not change with time, while *v*_*t*_ is the year fixed effect. ε_*i,t*_ is a random disturbance term.

Coefficients β_2_ and β_1_β_3_ are the focus of our study. $\beta_{2}^{m}$ reveals the direct spillover effect of the wife's retirement on the husband's cognitive health; $\beta_{1}^{m}\beta_{3}^{f}$ reveals the indirect spillover effect the wife's retirement on the husband's cognitive health. $\beta_{2}^{f}$ reveals the direct spillover effect of the husband's retirement on the wife's cognitive health; $\beta_{1}^{f}\beta_{3}^{m}$ reveals the indirect spillover effect the husband's retirement on the wife's cognitive health.

The identification of the equation (1) is faced with measurement bias arising from two main problems. (i) The endogeneity problem between retirement and cognitive health; and (ii) the problem of over-identification of the simultaneous equations model. For the second problem, this paper requires the use of three-stage least squares (3SLS) for estimation. The first problem is relatively difficult to solve.

To address the endogeneity problem between retirement and cognitive health, this paper draws on Lee and Lemieux (26) and Pique (27) to employ the Fuzzy Regression Discontinuity Design (FRD) method. Specifically, using the male and female statutory retirement age policies as instrumental variables for husbands' and wives' retirement status, respectively. Hence, the equations are constructed for the relationship between the retirement status of husbands and wives and the instrumental variables.


(2) $$\begin{array}{r} R_{i,t}^{m}=\gamma_{0}^{m}+\gamma_{1}^{m}D_{i,t}^{m}+\gamma_{2}^{m}X_{i,t}^{m}+\gamma_{3}^{m}g_{i,t}^{m}\left(age_{i,t}^{m}\right)\\ +\mu_{i}^{m}+v_{t}^{m}+\epsilon_{i,t}^{m} \end{array}$$

(3) $$\begin{array}{r} R_{i,t}^{f}=\gamma_{0}^{f}+\gamma_{1}^{f}D_{i,t}^{f}+\gamma_{2}^{f}X_{i,t}^{f}+\gamma_{3}^{f}g_{i,t}^{f}\left(age_{i,t}^{f}\right)+\mu_{i}^{f}\\ +v_{t}^{f}+\epsilon_{i,t}^{f} \end{array}$$

$D_{i,t}^{m}$ and $D_{i,t}^{f}$ are the instrumental variables for the husband's and wife's retirement status, respectively. They are determined by the difference between the actual age of individual and the statutory retirement age. The actual age of the husband is represented by $age_{i,t}^{m}$, The actual age of the wife is represented by $age_{i,t}^{f}$. When the actual age at time *t* is greater than or equal to the statutory retirement age, then $D_{i,t}^{m}=$ 1 ($D_{i,t}^{f}=$ 1); otherwise, $D_{i,t}^{m}=$ 0 ($D_{i,t}^{f}=$ 0). $g_{i,t}^{m}\left(age_{i,t}^{m}\right)$ and $g_{i,t}^{f}\left(age_{i,t}^{f}\right)$ are polynomials of the husband's and wife's age, respectively. Referring to previous literature (28), this paper sets $g_{i,t}^{m}\left(age_{i,t}^{m}\right)$ and $g_{i,t}^{f}\left(age_{i,t}^{f}\right)$ as second-order polynomials for the ages of husbands and wives. The second-order polynomials are included to construct non-linear relationships for regression to prevent model setting bias. And the remaining variables have the same meaning as in equation (1).

Finally, the fitted values of $R_{i,t}^{m}$ and $R_{i,t}^{f}$ obtained from equations (2) and (3) are substituted into the equation (1) respectively, thus solving the endogeneity problem between individual retirement, spouse's retirement and individual cognitive health to some extent.

#### Setting the Mediating Effect Model

To effectively reveal the mechanisms through which the spouse's retirement impacts on the individual's cognitive health, we set the following recursive model to test the effect of the mediating variables, which is based on the testing method proposed by Wen and Ye (29):


(4) $$\begin{array}{r} C_{i,t}=\alpha_{0}+\alpha_{1}R_{i,t}+\alpha_{2}R_{j,t}+\alpha_{3}X_{i,t}+\mu_{i}\\ +v_{t}+\varepsilon_{i,t} \end{array}$$

(5) $$\begin{array}{r} mediator_{i,t}=\rho_{0}+\rho_{1}R_{i,t}+\rho_{2}R_{j,t}+\rho_{3}X_{i,t}+\mu_{i}\\ +v_{t}+\varepsilon_{i,t} \end{array}$$

(6) $$\begin{array}{r} C_{i,t}=\sigma_{0}+\sigma_{1}mediator_{i,t}+\sigma_{2}R_{i,t}+\sigma_{3}R_{j,t}+\sigma_{4}X_{i,t}+\mu_{i}\\ +v_{t}+\varepsilon_{i,t} \end{array}$$

where *i* ≠ *j*, *i* denotes an individual and *j* denotes their spouse. *mediator*_*i,t*_ represents the mechanism variable. The remaining variables have the same meaning as in equation (1).

The focused parameters are (ρ_2_ × σ_1_) and σ_3_. The (ρ_2_ × σ_1_) captures the mediating effect of the mechanism variables, and the σ_3_ captures the direct effect of the spouse's retirement on the individual cognitive health.

We identify the mediating effect by the following steps: (1) Testing α_2_. If α_2_ is significant, it means that the spouse's retirement has a spillover effect on the individual cognitive health, and it makes sense to explore the underlying mechanisms. (2) Testing ρ_2_. If ρ_2_ is significant, it means that the spouse's retirement could influence the mechanism variables. (3) Testing σ_1_ and (ρ_2_ × σ_1_). If both σ_1_ and ρ_2_ are significant, it means (ρ_2_ × σ_1_) is significant, and that the spouse's retirement affects an individual cognitive health by the mechanism variables; if at least one of σ_1_ and ρ_2_ is not significant, the significance of (ρ_2_ × σ_1_) needs to be tested by Sobel *Z*-test. (4) Testing σ_3_. If σ_3_ is significant, it means that there is direct effect of the spouse's retirement on individual's cognitive health. (5) Judging the type of the mediating effect. If both (ρ_2_ × σ_1_) and σ_3_ are significant and in the same direction, there is a complementary mediating effect; if (ρ_2_ × σ_1_) and σ_3_ are significant but in the different direction, there is a competitive mediating effect; if (ρ_2_ × σ_1_) is significant but σ_3_ is not, there is a full mediating effect; if σ_3_ is significant but (ρ_2_ × σ_1_) is not, there is none mediating effect.

Notably, when the spouse's cognitive health is used as the mediator, the mediating effect (ρ_2_ × σ_1_) actually corresponds to the indirect spillover effect ($\beta_{1}^{m}\beta_{3}^{f}$ or $\beta_{1}^{f}\beta_{3}^{m}$), and the direct effect σ_3_ corresponds to the direct spillover effect ($\beta_{2}^{m}$ or $\beta_{2}^{f}$).

## Results

### Basic Descriptive Analysis

Table 1 shows that husbands' cognitive health is generally healthier than that of wives, with a mean of 0.315 for husbands' cognitive health and 0.133 for wives' cognitive health. The average age of husbands is 58 years and the average age of wives is 49 years. The percentage of the retired in the sample of husbands is 37.4% and the percentage of the retired in the sample of wives is 57.9%. From the data on the predetermined variables, it can be seen that the majority of dual-earner couples has the following characteristics: urban-hukou, primary and middle school education level, family size ranges from 3 to 4 members, possessing no more than one house, participated in medical insurance, and mainly cared by their spouses in case of illness.

**Table 1:** Descriptive statistics.

**Variables**		**Individual**		**Spouse**		**Husband**		**Wife**	
		**Mean**	**S.D**.	**Mean**	**S.D**.	**Mean**	**S.D**.	**Mean**	**S.D**.
**Explained variable**									
Cognitive health		0.224	0.735	0.226	0.733	0.315	0.684	0.133	0.772
**Core explanatory variables**									
Age		57.148	5.338	57.145	5.325	58.176	5.242	49.121	5.234
Spouse retirement status		0.468	0.499	0.469	0.499	0.579	0.494	0.374	0.484
Retirement status		0.469	0.499	0.468	0.499	0.374	0.484	0.579	0.494
**Predetermined variables**									
Urban-hukou		0.937	0.242	0.938	0.242	0.939	0.24	0.936	0.244
Illiterate/semi-literate		0.156	0.363	0.141	0.348	0.11	0.312	0.203	0.402
Primary and middle school education		0.495	0.500	0.502	0.500	0.516	0.500	0.474	0.499
High school and above education		0.259	0.438	0.267	0.443	0.257	0.437	0.262	0.44
Family size		3.605	1.652	3.605	1.652	3.601	1.655	3.608	1.649
Possess more than one house		0.323	0.468	0.323	0.468	0.323	0.468	0.324	0.468
Participated in medical insurance		0.878	0.327	0.877	0.329	0.885	0.319	0.871	0.336
Mainly cared by spouse in case of illness		0.673	0.469	0.673	0.469	0.716	0.451	0.630	0.483
**Mechanism variables**									
Social interaction	Spouse cognitive health	0.226	0.733	0.224	0.735	0.133	0.772	0.315	0.684
Family resources	Household income per capita	9.662	1.008	9.662	1.008	9.664	1.003	9.66	1.013
	The share of housework undertaken by the wife	0.683	0.246	0.683	0.246	0.683	0.246	0.684	0.247
Health lifestyle	Cigarette amounts a day	5.915	11.563	5.908	11.535	10.821	13.954	0.477	3.136
	Drink alcohol frequently	0.900	1.725	0.901	1.725	0.927	1.57	0.874	1.867
	Exercise frequency	3.769	3.444	3.776	3.445	3.909	3.482	3.630	3.399

As for the mechanism variables, the spouse's cognitive health is described in the above paragraph. The mean value of the household income per capita after taking the logarithm is 9.66, that is, the mean value of household income per capita is around 16,000. The data of the share of housework undertaken by the wife shows that the wife is the main undertaker of housework, and the proportion of the wife's daily housework time in the total housework time of husband and wife is generally 68%. Husbands' lifestyle is much less healthy than their wives', with husbands being more likely than wives to smoke daily and to drink frequently. About exercise frequency, the data show husbands and wives both exercise 3-4 times per week.

**Figure 2:**
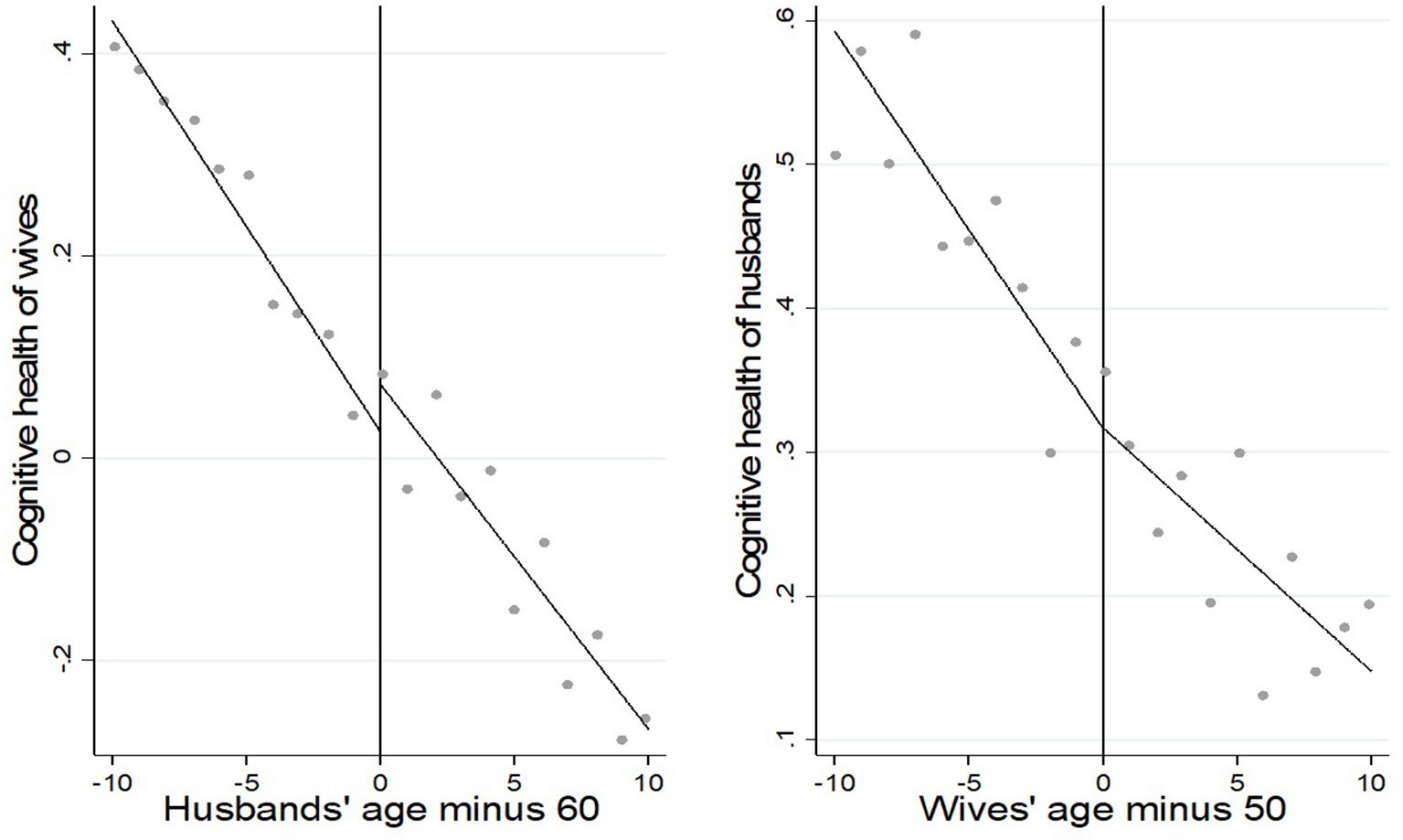
The spouse's retirement and the individual's cognitive health.

We give a graphical representation of the spousal retirement effect on the individual's cognitive health (see Figure 2). The figure shows that the individual's cognitive health declines with age. In the left-hand graph of Figure 2, there is a significant downward jump in the wife's cognitive health after the husband retires. This means a significant negative spillover effect of the husband's retirement on the wife's cognitive health. In contrast, the husband's cognitive health has no significant jump after the wife's retirement in the right-hand graph of Figure 2, which may indicate a weak effect of the wife's retirement on the husband's cognitive health.

### Basic Regression Results

Table 2 presents the estimates of the effect of the spouse's retirement on individual cognitive health, including the estimates of the ordinary least squares (OLS) model and the corresponding robust standard errors. In particular, column (1) lists the estimates when the spouse's retirement and control variables are added; column (2) lists the estimates when the spouse's retirement, own retirement, and control variables are controlled; column (3) lists the estimates when the spouse's retirement, own retirement, spouse cognitive health and control variables are controlled. The estimates in columns (1)-(3) show that after a series of characteristic variables are controlled, the parameter estimates of the spouse's retirement are still positive, but only is the result of column (3) significant at the 5% significance level.

**Table 2:** OLS regression results.

**Variable**	**Explained variable: individual cognitive health**		
	**(1)**	**(2)**	**(3)**
Spouse's retirement	0.066 (0.041)	0.060 (0.044)	0.089^**^ (0.044)
Own retirement	—	−0.113^***^ (0.010)	−0.106^**^ (0.052)
Spouse cognitive health	—	—	0.171^***^ (0.018)
Age	−0.009^***^ (0.003)	−0.006^***^ (0.002)	−0.006^***^ (0.002)
Age^2^/100	0.068^*^ (0.035)	0.045 (0.043)	0.038 (0.041)
_cons	0.892^***^ (0.090)	0.945^***^ (0.108)	0.815^***^ (0.107)
Control variables	Yes	Yes	Yes
Individual fixed-effects	No	No	No
Year and province dummy variables	Yes	Yes	Yes
*N*	9,684	9,636	9,624
R-squared	0.654	0.662	0.685

*Standard errors are in parentheses;*

* *p < 0.1*,

** *p < 0.05*,

*** *p < 0.01. Robust standard errors are reported*.

In column (1), the result shows there is not a spillover effect of the spouse's retirement on individual cognitive health. In column (2), the result shows the spillover effect of the spouse's retirement is not significant, but individual own retirement reduces cognitive health at the 1% significance level with an effect of −0.113. In column (3), each unit increase in the spouse's retirement contributed to a 0.089 unit increase in individual cognitive health. Individual own retirement significantly reduces the probability of improved cognitive health. Improvement in spousal cognitive health significantly increased the probability of improvement in individual cognitive health, in other words, there is a positive social interaction effect between couples in terms of cognitive health. It can be seen from the above that OLS regression results are not robust or reliable because endogenous problems and individual fixed-effects are ignored.

### Results of the Fuzzy Regression Discontinuity Design

Table 3 presents the estimates of the FRD model with panel individual fixed-effects model. The results of the first stage regression show that the F-statistics of the weak instrumental variable was much >10, indicating that the selected instrumental variable was highly correlated with the endogenous explanatory variables. Therefore, the possibility of a weak instrumental variable can be ruled out. The results of the second stage regression show that the coefficients of the spouse's retirement are all negative (at the 1% level of significance), and this remained consistent in columns (1)-(2). This indicates that there is a significant adverse effect of the spouse's retirement on individual cognitive health after potential endogeneity issues are overcome using the FRD model with panel individual fixed-effects model. In column (5), the spouse's retirement, own retirement, spouse cognitive health and control variables are all controlled and estimated using the FE-3SLS method. The result shows that the spillover effect of the spouse's retirement on individual cognitive health is −0.213 at the 5% significance level, the effect of individual retirement on own cognitive health is −0.819 at the 1% significance level, and the improvement in cognitive health of one spouse would lead to 60.2% improvement in the cognitive health of the other spouse.

**Table 3:** FRD regression results.

**Variable**	**Explained variable: individual cognitive health**	
	**(1)**	**(2)**
Spouse's retirement	−0.161^***^ (0.015)	−0.213^**^ (0.095)
Own retirement	−0.748^***^ (0.094)	−0.819^***^ (0.063)
Spouse cognitive health	—	0.602^***^ (0.052)
Age	−0.005^*^ (0.003)	−0.048^***^ (0.007)
Age^2^/100	0.028 (0.037)	−0.228^***^ (0.041)
_cons	0.832^***^ (0.095)	1.161^***^ (0.117)
Control variables	Yes	Yes
Individual fixed-effects	Yes	Yes
Year and province dummy variables	Yes	Yes
N	3624	3620
R-squared	0.363	−0.319
The first stage F value	85.81	79.32

* *p < 0.1*,

** *p < 0.05*,

*** *p < 0.01. Standard errors are in parentheses; Robust standard errors are reported*.

Combined with the above analysis, this paper finds that there is a significant negative spillover effect of the spouse's retirement on individual cognitive health, and individual retirement also has a negative impact on their own cognitive health. In addition, there is a positive social interaction effects of the couple's cognitive health, that is, when one spouse cognitive health decreases, the other spouse cognitive health also decreases.

### Results of the Heterogeneity by Gender

Taking into account the inconsistency of the legal retirement age between male and female in China, and the traditional division of domestic chores, this paper investigates the gender heterogeneity in the spillover effect of the spouse's retirement on individual cognitive health.

The results in Table 4 show that the spouse's retirement has a significantly negative spillover effect on the cognitive health of both husbands and wives. The gender heterogeneity is reflected in the stronger negative spillover effect of husbands' retirement than wives' retirement. Specifically, the spillover effect of husbands' retirement on wives' cognitive health is significant at −0.503, while the spillover effect of wives' retirement on husbands' cognitive health is significant at −0.312.

**Table 4:** Heterogeneity by gender.

**Variable**	**(1) Husband**	**(2) Wife**
Spouse's retirement	−0.312^**^ (0.147)	−0.503^***^ (0.092)
Own retirement	−0.681^***^ (0.122)	−1.187^***^ (0.121)
Spouse cognitive health	0.235^***^ (0.027)	0.528^***^ (0.022)
Control variables	Yes	yes
Individual fixed-effects	Yes	yes
Year and province dummy variables	Yes	yes
N	1,764	1,756
R-squared	0.517	0.604
The first stage F value	71.83	63.48
Direct spillover effect	−0.312^**^ (0.147)	−0.503^***^ (0.092)
Indirect spillover effect	−0.279^***^ (0.043)	−0.360^***^ (0.066)

* *p < 0.1*,

** *p < 0.05*,

*** *p < 0.01*.

*Standard errors are in parentheses; Robust standard errors are reported. The significance of the indirect spillover effect is obtained by Sobel Z-test*.

Besides, as shown in Table 4, the individual retirement has a significantly negative spillover effect on their own cognitive health, and there is a significantly positive social interaction effect between the cognitive health of the couples. Wives' retirement decreases their own cognitive health by 1.187, while husbands' retirement decreases their own cognitive health by 0.681. The results of the social interaction effect demonstrate that husbands' cognitive health is impacted at 0.235, while wives' is impacted at 0.528.

Based on the results in the first three rows of Table 4, the direct and the indirect spillover effects of the spouse's retirement on individual cognitive health are calculated separately, and the results are shown in the last two rows of Table 4. It can be concluded that the direct spillover effect of wives' retirement on husbands' cognitive health is significantly −0.312, and that of husbands' retirement on wives' cognitive health is significantly −0.503. The indirect spillover effect of wives' retirement on husbands' cognitive health is significant at −0.279, and the indirect spillover effect of husbands' retirement is significant at −0.360.

In summary, both the direct and the indirect spillover effects of the spouse's retirement are significantly negative, with the direct spillover effect dominating. Compared with the results of wives' retirement, the direct and the indirect spillover effects of husbands' retirement on wives' cognitive health are stronger. This implies that women's cognitive health is more likely to be adversely affected by their spouses' retirement.

### Specification Test

Since the validity of FRD results requires the following two points: (1) the running variable (age) must satisfy a random distribution and cannot be manipulated by respondents. (2) the predetermined variables cannot change discontinuously at the cutoff point. In the specification test, we check the two requirements.

#### Density Distribution of Age

**Figure 3:**
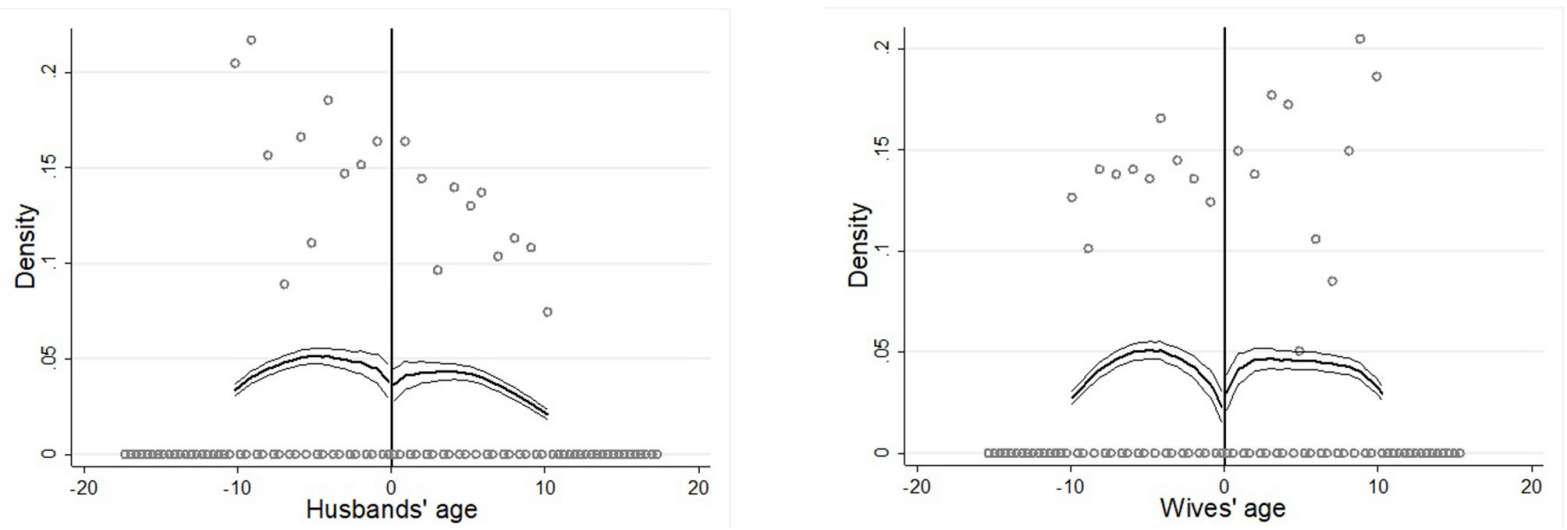
Density distribution of age.

In response to the first requirement, the density distribution of age is examined for continuity following the McCrary (30) method. As shown in Figure 3, the distribution of ages does not jump at the cutoff. Therefore, it can be judged that the ages are randomly distributed within the specified window width. The first requirement of the FRD method is satisfied.

#### Continuity Test of Predetermined Variables

The result of the second requirement is shown in Table 5. As we can see, the coefficients of the six predetermined variables are not significant. This means that these predetermined variables do not jump at the cutoff. Therefore, the above empirical results regarding the spillover effect of the spouse's retirement on individual cognitive health are valid.

**Table 5:** Continuity test of predetermined variables.

**Variable**	**Education level**	**Urban- hukou**	**Possess more than one house**	**Family size**	**Participated in medical insurance**	**Mainly cared by spouse in case of illness**
Husband	0.205 (0.178)	0.142 (0.326)	0.059 (0.055)	−0.187 (0.832)	0.179 (0.209)	0.031 (0.078)
Wife	0.180 (0.823)	0.064 (0.138)	0.167 (0.265)	−0.263 (0.347)	0.053 (0.123)	0.037 (0.052)

### Robustness Check

#### Sensitivity Test of Window Width

In the sensitivity test of window width, this paper is reduced from the original [−10, 10] to [−6, 6] and [−8, 8]. The results are shown in Table 6. The results remain consistent with Table 4 regardless of the variation in window width. Hence, the conclusions obtained in this paper are robust.

**Table 6:** Sensitivity test of window width.

**Variable**	**Window width = 6**		**Window width = 8**	
	**Husband**	**Wife**	**Husband**	**Wife**
Spouse's retirement	−0.412^**^ (0.174)	−0.806^***^ (0.096)	−0.432^**^ (0.201)	−0.853^***^ (0.087)
Own retirement	−0.242^**^ (0.114)	−2.011^***^ (0.108)	−0.179^*^ (0.102)	−1.998^***^ (0.097)
Spouse cognitive health	0.107^***^ (0.033)	0.131^***^ (0.043)	0.128^***^ (0.030)	0.140^***^ (0.038)
Control variables	Yes	Yes	Yes	Yes
Individual fixed-effects	Yes	Yes	Yes	Yes
Year and province dummy variables	Yes	Yes	Yes	Yes
*N*	788	841	1,422	1,428

* *p < 0.1*,

** *p < 0.05*,

*** *p < 0.01. Standard errors are in parentheses; Robust standard errors are reported*.

#### Placebo Test for Cutoffs

Table 7 shows placebo tests at other nearby cutoff points, −3, +3. In the placebo test for cutoffs-3, the instrumental variable for husband's retirement is 57 years for men and 47 years for women; and in the placebo test for cutoffs+3, the instrumental variable for husband's retirement is 63 years for men and 53 years for women. As expected, there is no effect at other cutoff points, and the results in this paper are robust.

**Table 7:** Placebo test for cutoffs.

**Variable**	**cutoffs-3**		**cutoffs+3**	
	**Husband**	**Wife**	**Husband**	**Wife**
Spouse's retirement	−0.182 (0.836)	−0.108 (0.092)	−0.045 (0.096)	−0.101 (0.089)
Own retirement	0.413 (0.924)	−0.343 (0.233)	0.239 (0.153)	−0.182 (0.206)
Spouse cognitive health	0.107^***^ (0.033)	0.106^**^ (0.043)	0.128^***^ (0.030)	0.140^***^ (0.038)
Control variables	Yes	Yes	Yes	Yes
Individual fixed-effects	Yes	Yes	Yes	Yes
Year and province dummy variables	Yes	Yes	Yes	Yes

* *p < 0.1*,

** *p < 0.05*,

*** *p < 0.01*.

*Standard errors are in parentheses; Robust standard errors are reported*.

## Mechanism Testing

In this section, we empirically test three potential mechanisms. Where the indirect spillover effect may happen through social interaction mechanism, the direct spillover effect may occur through the two mechanisms of family resources and health lifestyle. The results are shown in Table 8.

**Table 8:** Results of the mediating effects.

**Type**	**Mechanisms**	**Mediating effects**	**Direct effects**	**Types of mediating effects**
**Panel A: Husband cognitive health**				
Social interaction	Social interaction effects of the couple's cognition	−0.279^***^	−0.312^**^	Complementary
Family resources	Household income per capita	−0.005^***^	−0.009^**^	Complementary
	The share of housework undertaken by the wife	0.008^***^	−0.097^*^	Competitive
Health lifestyle	Cigarette amounts a day	0.071	−0.018^***^	None
	Drink alcohol frequently	−0.027^**^	−0.213^**^	Complementary
	Exercise frequency	0.038^**^	−0.160^**^	Competitive
**Panel B: Wife cognitive health**				
Social interaction	Social interaction effects of the couple's cognition	−0.360^***^	−0.503^***^	Complementary
Family resources	Household income per capita	−0.713^***^	−0.026	Full
	The share of housework undertaken by the wife	0.001	−0.273^**^	None
Health lifestyle	Cigarette amounts a day	−0.044	−0.054^*^	Complementary
	Drink alcohol frequently	0.002	−0.039^**^	None
	Exercise frequency	0.108^***^	−0.352^***^	Competitive

* *p < 0.1*,

** *p < 0.05*,

*** *p < 0.01*.

*Standard errors are in parentheses; Robust standard errors are reported. The significance of the mediating effect is obtained by Sobel Z-test*.

Combining equation (1) with equations (4)-(6), we can know that the mediating effect of social interaction mechanism actually corresponds to the indirect spillover effect in this paper. It is stated again here mainly to ensure the completeness of the mechanism analysis, and to distinguish the mechanisms of the direct spousal spillover effects from those of the indirect spousal spillover effects.

### Testing Social Interaction Mechanism

In social interaction mechanism, the spouse's cognitive health is the mediator. The results show that the wife's retirement has a negative effect on her own cognitive health, which in turn leads to a decline in her husband cognitive health. Through the social interaction effects of the cognition, the husband's retirement also leads to the wife's cognitive decline.

### Testing Family Resources Mechanism

The results on family resources in Table 8 show that wives' retirement leads to a decline in household income per capita, and husbands' cognitive health is affected by the decline in income. On the other hand, wives will spend more time on housework after retirement, and contribute more to housework. As a result, husbands receive more care and has more time for recreation and leisure. The negative spillover effect of wives' retirement on husbands' cognitive health is mitigated by the mediating variable of the share of housework undertaken by the wife.

Husbands' retirement results in a significant decrease in household income per capita, thus adversely affecting wives' cognitive health. However, the increase in husbands' contribution to housework does not have a significant mediating effect on wives' cognitive health. This may be due to the fact that although the husbands' contribution to housework increases, the wives still take on more housework, which is caused by the traditional division of domestic responsibilities between husbands and wives.

### Testing Health Lifestyle Mechanism

In terms of health lifestyle, wives' retirement significantly increases husbands' cigarette amounts a day, probability of drink alcohol frequently and exercise frequency. The increase in cigarette amounts a day does not significantly affect the husbands' cognitive health, and the increased probability of drink alcohol frequently decreases husbands' cognitive health. But the increased exercise frequency mitigates the decline trend of husbands' cognition.

Husbands' retirement also has a significant effect on wives' health lifestyle. Husbands' retirement increases wives' exercise frequency, but does not have a significant effect on wives' cigarette amounts a day or drink alcohol frequently. The increase in wives' exercise frequency improves cognitive health.

In summary, retirement not only has a negative effect on one's own cognitive health, but also has a negative effect on the cognitive health of the spouse through the social interaction effect of the couple's cognition. In addition, the spouse's retirement also has a negative spillover effect on individual cognitive health due to a decrease in household income per capita. The spouse's retirement can also bring about changes in individuals' health lifestyle. Wives' retirement has a negative spillover effect on husband cognitive health through an increase in the probability of frequent drinking. The share of housework undertaken by the wife and exercise frequency mitigate the negative spillover effect of wives' retirement on husband cognitive health to some extent, while the negative spillover effect of husbands' retirement on wife cognitive health is only mitigated by an increase in exercise frequency.

## Conclusions

Few studies measured the association between spouse's retirement and individual cognitive health among dual-earner couples from the perspective of the social interaction. This paper makes up for this and considers the gender heterogeneity. Using the 2010-2018 China Family Panel Studies data, we find that the spouse's retirement has a significant negative spillover effect on the individual cognitive health, which consists of the negative direct spillover effect and the negative indirect spillover effect. And the gender heterogeneity analysis indicate that the adverse effect of the husband's retirement is stronger than that of the wife's retirement. To analyze the underlying mechanisms, the mediating effects model are used. The results show that there is a significant positive social interaction effect of the couple's cognitive health. Besides, the decline in household income per capita brought about by the wife's retirement and the increase in the probability of frequent alcohol consumption by the husband's retirement are responsible for the husband's decreased cognitive health. The decline in household income per capita also is the channel through which the negative spillover effect of the husband's retirement on the wife cognitive health.

Our results contain abundant policy implications. For example, delaying the retirement age is beneficial to slowing cognitive decline, and government should take a forward-looking perspective to make the public aware of its necessity and gain public understanding and support. Furthermore, government should design the complementary measures to maximize and increase the beneficial effects of delayed retirement on middle-aged and elderly people and their families, and to avoid or reduce the adverse effects. Specifically, health preventive policies focusing on the family should be carried out, and the cultural products and healthcare services suitable for retirees should be provided. The discrimination in age and gender in labor market should be eliminated as far as possible, so as to support and encourage individuals, women in particular, to delay retirement or re-employment.

There are several limitations in this paper. First, this paper does not take into account transitions between different family structures (e.g., death, divorces and re-marriages) that may affect the retirement decision and the optimal investments in health. Second, the data about career choice lacks so that heterogeneity analysis by career in this article is missing. Third, based on the FRD method, our results are deficient in external validity. However, in the absence of randomization of the age of retirement, we believe that this is as close as we can estimate a causal effect. Finally, the mechanism testing does not address the potential endogeneity of the mediators since it is difficult to find convincing instruments for each of the mediating variables. For example, household income actually is endogenous to the spouse's cognitive health. The endogeneity between household income and spouse's cognitive health could arise due to two sources: (i) reverse causality, i.e., declining cognitive health of the spouse can lead to a decrease in household income; and (ii) omitted variable bias, e.g., the cognitive ability of the self-disciplined people may be healthier, and their household may be wealthier. The positive endogeneity may imply the true results are greater than our results.

## Data Availability Statement

The original contributions presented in the study are included in the article/supplementary material, further inquiries can be directed to the corresponding author/s.

## Ethics Statement

The studies involving human participants were reviewed and approved by the Biomedical Ethics Review Committee of Peking University approved CFPS, and all participants were required to provide written informed consent. The ethical approval number was IRB00001052-14010. The patients/participants provided their written informed consent to participate in this study.

## Author Contributions

XX contributed to the study design, analyzed the data, and took the lead in the manuscript writing. RL designed the study and proposed amendments. HY helped in the writing of the final draft of the manuscript. All authors have revised the manuscript and approved the submitted version.

## Funding

This study was supported by the Humanities and Social Sciences Fund of the Ministry of Education (Grant No. 19YJC790167).

## Conflict of Interest

The authors declare that the research was conducted in the absence of any commercial or financial relationships that could be construed as a potential conflict of interest.

## Publisher's Note

All claims expressed in this article are solely those of the authors and do not necessarily represent those of their affiliated organizations, or those of the publisher, the editors and the reviewers. Any product that may be evaluated in this article, or claim that may be made by its manufacturer, is not guaranteed or endorsed by the publisher.

## Footnotes

^1^“Stable Unit Intervention Value Assumption (SUTVA)” has two components: (1) the assumption that the potential outcome of any individual is not disturbed by the treatment outcome of other individuals; (2) the assumption that the relationship between the treatment state received by each individual and the potential outcome is clear and unique, independent of the allocation mechanism (16).

^2^In 2011 labor force in China included 359 million people in urban areas and 405 million people in rural areas (20).

^3^This question also specifies whether the respondent is retired but rehired. As a result, our control group also includes those who are re-employed after retirement.
